# A Systematic Review and Meta-Analysis of Randomized Controlled Trials With Novel Hormonal Therapies for Non-Metastatic Castration-Resistant Prostate Cancer: An Update From Mature Overall Survival Data

**DOI:** 10.3389/fonc.2021.700258

**Published:** 2021-06-08

**Authors:** Martina Maggi, Stefano Salciccia, Francesco Del Giudice, Gian Maria Busetto, Ugo G. Falagario, Giuseppe Carrieri, Matteo Ferro, Angelo Porreca, Giovanni Battista Di Pierro, Vittorio Fasulo, Viviana Frantellizzi, Giuseppe De Vincentis, Ettore De Berardinis, Alessandro Sciarra

**Affiliations:** ^1^ Department of Maternal-Infant and Urological Sciences, Sapienza Rome University, Policlinico Umberto I, Rome, Italy; ^2^ Department of Urology and Renal Transplantation, University of Foggia, Policlinico Riuniti, Foggia, Italy; ^3^ Department of Urology, European Institute of Oncology (IEO), Milan, Italy; ^4^ Department of Urology, Veneto Institute of Oncology (IOV) IRCCS, Padua, Italy; ^5^ Department of Urology, Istituto Clinico Humanitas IRCCS-Clinical and Research Hospital, Milan, Rozzano, Italy; ^6^ Department Of Maternal-Infant And Urological Sciences, Sapienza Rome University, Policlinico Umberto I, Rome, Italy

**Keywords:** prostate neoplasm, non-metastatic castration-resistant prostate cancer, hormonal therapy, overall survival, adverse events, metastasis

## Abstract

**Introduction:**

To get better insight into the management of non-metastatic castration-resistant prostate cancer (M0 CRPC), in this meta-analysis and review we aimed to present an updated evaluation of the efficacy and safety of novel hormonal therapies (nHT) for M0 CRPC according to final analyses with mature overall survival (OS) and safety data.

**Methods:**

We analyzed metastasis-free survival (MFS), OS, time to prostate-specific antigen (PSA) progression, second-line therapies data, adverse events (AEs), including all AEs, serious AEs (SAEs), AEs leading to discontinuation of trial regimen, AEs leading to death, fatigue, dizziness, cardiovascular events, and fractures; moreover, we evaluated the impact of PSA doubling time (PSA-DT), Eastern Cooperative Oncology Group (ECOG) score, use of bone-targeted therapy, lymph lodes (LN) status, and prior HT on final OS data. A comparison among the placebo arms of the included trials in terms of survival and safety profiles was assessed.

**Results:**

According to the pooled analysis with updated and mature OS data, OS was significantly improved with nHT compared to placebo (hazard ratio (HR)= 0.74, 95% confidence interval (CI)= 0.66–0.84). nHT significantly improved OS over placebo across all pre-specified subgroups. Subgroup analysis revealed a greater OS benefit in patients with PSA-DT >6 months than ≤6 months (HR= 0.69 versus HR= 0.75), ECOG 0 than 1 (HR= 0.70 versus HR= 0.80), N1 disease than N0 (HR= 0.61 versus HR= 0.78), and in those receiving bone-targeted therapy (HR= 0.65 versus HR= 0.74), and a comparable OS by number of prior HT (HR= 0.75 versus HR= 0.76, for HT= 1 and ≥2); yet, differences between pre-specified subgroups were not significant (all p> 0.05). Overall, the nHT arm was significantly associated with higher rates of AEs, when compared with the placebo arm. The long-term analysis showed a worse safety profile with nHT than the interim analysis.

**Conclusions:**

According to final analyses, nHT have shown to improve OS over placebo in the setting of high-risk M0 CRPC. The long-term analysis showed a worse safety profile with nHT than the interim analysis, whit distinct profiles among different nHT. The lack of survival data regarding second-line therapies remains a major issue.

## Introduction

Androgen deprivation therapy (ADT) is the basis of the medical treatment for advanced prostate cancer (PC), and for those men with early-stage PC who experience biochemical progression with a rising prostate-specific antigen (PSA) level after curative treatment (1–4). ADT can be achieved with either surgery (i.e. bilateral orchiectomy) or various agents (i.e. gonadotropin-releasing hormone (GnRH) agonists, GnRH antagonists and anti-androgens), and despite it is initially effective, eventually the majority of cases will experience progression to a castration-resistant PC (CRPC) (5). According to the European Association of Urology (EAU) guidelines, CRPC can be defined as castrate serum testosterone (<50 ng/dL or 1.7 nmol/L) plus either biochemical or radiological progression (6). The status with a progressive rising PSA levels, in a low testosterone environment, and in the absence of detectable metastasis on conventional imaging, is known as non-metastatic CRPC (M0 CRPC), whose prevalence has been estimated to about 10% of PC in Europe (7, 8). It has also been observed that metastasis-free survival (MFS) in this setting is 25 to 30 months, and that about one-third will develop visible bone metastases within 2 years (9).

Since metastatic CRPC (mCRPC) is fatal, with a median survival of approximately 3 years, currently prolonging as long as possible the M0 status by delaying the onset of metastasis and the need of subsequent treatments -with the related side effects- is a major treatment goal in M0 CRPC. Until recently, no approved systemic therapies existed for these cases, and observation in the context of on-going ADT was the standard of care. This scenario changed in 2018, with the sequential approvals of novel hormonal therapies (nHT) (i.e. Enzalutamide, Apalutamide, and Darolutamide), after 3 phase III randomized controlled trials (RCTs) were carried out comparing these drugs to placebo (i.e. treatment with the sole on-going ADT) in high-risk M0 CRPC cases (i.e. M0 CRPC cases with a PSA doubling time (PSA-DT) of ≤ 10 months) (10–12). Although all 3 trials met their primary endpoint (i.e. MFS), skepticism was raised regarding MFS as a clinically relevant endpoint, and whether it would reflect an improved OS. Indeed, at the primary analyses, none of the studies showed an OS benefit due to immature data (6). Updated data regarding OS were presented at the 2020 American Society of Clinical Oncology (ASCO) Annual Meeting, and then recently published, showing clearer results (13–15). Moreover, since different drugs appeared to be comparable in terms of oncological profile, and in view of the long-term treatment with these agents in asymptomatic patients, the therapeutic choice should be based on safety profile. To date, no direct comparison among these compounds has been made.

To get better insight into the management of these cases, and to further guide the future choice among these novel compounds, in this meta-analysis and review we aimed to present an updated evaluation of the efficacy and safety of nHT for M0 CRPC cases according to last publications with mature OS and safety data; moreover, we sought to assess whether there existed differences in the placebo arms of the evaluated studies in terms of oncological outcomes and safety profiles, which could have influenced comparative results among nHT trials.

## Methods

### Objective

The primary aim of the present meta-analysis is to systematically analyze the current evidence on nHT for M0 CRPC cases.

In particular, in populations of M0 CRPC cases, we analyzed: MFS, OS, time to PSA progression, second-line therapies data, adverse events (AEs) (overall AEs and grade 3-4 AEs) including all AEs, serious AEs (SAEs), AEs leading to discontinuation of trial regimen, AEs leading to death, fatigue, dizziness, cardiovascular events, and fractures; moreover we evaluated the impact of PSA-DT (defined as the time required for the PSA level to double; ≤ versus >6 months), performance status (PS) (Eastern Cooperative Oncology Group (ECOG) score 0 versus 1), the use of bone-targeted therapy (yes versus no), lymph lodes (LN) status (N0 versus N1), and prior HT (1 versus ≥2) on updated OS data. A comparison among the placebo arms of the included trials in terms of survival and safety profiles was assessed.

### Search Strategy

We searched in the Medline and Cochrane Library database and the American Society of Clinical Oncology (ASCO) Meeting (search terms: “prostate neoplasm” AND “castration-resistant prostate cancer” AND “non metastatic” AND “hormonal therapy” OR “apalutamide” OR “darolutamide” OR “enzalutamide”), without language restriction from the literature from January 2009 to September 2020, following The Preferred Reporting Items for Systematic Review and Meta-analyses (PRISMA) guidelines (**Figure S1**, **Supplementary Material**) (16). Original and review articles were included and critically evaluated. Additional references were identified from reference lists of these articles.

### Selection of the studies and Inclusion Criteria

Entry into the analysis was restricted to data collected from original studies on RCTs including subjects with a diagnosis of M0 CRPC who subsequently underwent treatment with Apalutamide, Enzalutamide, or Darolutamide.

Two authors (MM; AS) independently screened the titles and abstracts of all articles using predefined inclusion criteria. The full-text articles were examined independently by three authors (MM; SS; VF) to determine whether or not they met the inclusion criteria. Then, two authors (VF; GB) extracted data from the selected articles. Final inclusion was determined by all investigators’ evaluation discussion.

The studies selected for inclusion met the following criteria: (I) M0 CRPC cases; (II) Apalutamide, Enzalutamide, or Darolutamide as the experimental agent; (III) the comparison with placebo arm (i.e. received the sole ADT). **Table S1**, **Supplementary Material**, shows inclusion criteria following the Population, Intervention, Comparison, Outcomes and Study design (PICOS) method.

Articles were excluded if: (I) multiple reports were published on the same population, (II) data provided were insufficient for the outcomes described in the aim section, (III) animal studies, (IV) non-randomized studies.

### Statistical Analysis

Risk of bias (RoB) for all included studies was evaluated using the Review Manager (RevMan) (Copenhagen: The Nordic Cochrane Centre, The Cochrane Collaboration) tool for the assessment of the methodological quality of trials (**Figure S2**, **Supplementary Material**).

Random effects meta-analysis of class-level effect of nHT versus placebo was performed using the inverse variance technique for meta-analysis of hazard ratios (HRs) for efficacy outcomes, and the Mantel-Haenszel method for meta-analysis of dichotomous data for AEs. To explore the pre-defined outcomes of interest, subgroup analysis was performed regarding differences in the PS (ECOG score 0 versus 1), the use of bone-targeted therapy (yes versus no), LN status (N0 *vs* N1) and PSA-DT (>6 months *vs* <6 months). To assess the variance distribution of the event rates (ERs) of survival and safety outcomes in the sole placebo arms, pooled ERs with 95% confidence intervals (CIs) were calculated.

Heterogeneity was evaluated by X^2^ Q test and I^2^ statistic (17). For the Q test, p <0.05 indicated significant heterogeneity; for the I^2^ statistics, an I^2^ value >50% was considered significant. Our results are graphically displayed as forest plots, with HR with 95% CIs for the time-to-event variables, and odds ratio (ORs) or event rates (ERs) with 95% CIs for the dichotomous variables. Due to the small numbers of the included trials, no publication bias was estimated. Calculations were accomplished using RevMan version 5.4 (Copenhagen: The Nordic Cochrane Centre, The Cochrane Collaboration) and Stata version 16.1 (Stata Corporation, College Station, TX, USA). A p value of <0.05 was regarded as statistically significant, and all tests were two-sided.

## Results

### Search Results

The search strategy identified 2576 potentially relevant studies; after removing the duplicates, 1247 studies were screened of which 1224 were excluded based on title and abstract. For the remaining 23 studies, the full texts were obtained. The PRISMA flow diagram is presented in **Figure S1**, **Supplementary Material**. In total 3 studies fulfilled the inclusion criteria and were included in the final analysis (**Table 1**).

**Table 1:** Patients baseline characteristics in the 3 included studies by treatment group [number of cases (%), and median (range)].

Variable	ARAMIS (12)		PROSPER (10)		SPARTAN (11)	
	(n= 1509)		(n= 1401)		(n= 1207)	
**Arm**	DAROLUTAMIDE	PLAC	ENZALUTAMIDE	PLAC	APALUTAMIDE	PLAC
**Patients**, *n°*	955	554	933	468	806	401
**Age** (years)*, median (range)*	74 (48–95)	74 (50–92)	74 (50–95)	74 (53–92)	74 (48–94)	74 (52–97)
Follow-up * (months)*, median*	29.0		48.0		52.0	
**Time from initial diagnosis** (months)*, median*	86.2	84.2	n.s.	n.s.	95.4	94.2
**Total PSA level** (ng/mL)*, median (range)*	9.0 (0.3–858.3)	9.7 (1.5–885.2)	11.1 (0.8–1071.1)	10.2 (0.2–467.5)	7.8	8.0
**Testosterone level** (nmol/L)*, median (range)*	0.6 (0.2–25.9)	0.6 (0.2–7.3)	n.s.	n.s.	0.8 (0.3–3.1)	0.8 (0.3–2.8)
**PSA-DT** *, n° (%)*						
≤6 months	667 (70)	371 (67)	715 (77)	361 (77)	576 (72)	284 (71)
>6 months	288 (30)	183 (33)	217 (23)	107 (23)	230 (29)	117 (29)
*median* (months)	4.4	4.7	3.8	3.6	4.4	4.5
**LN status**, *n° (%)*						
**N0**	792 (83)	396 (71)	n.s.	n.s.	673 (84)	336 (84)
**N1**	163 (17)	158 (29)			133 (17)	65 (16)
**PS ECOG score**, *n° (%)*						
**0**	650 (68)	391 (71)	747 (80)	382 (82)	623 (77)	311 (78)
**1**	305 (32)	163 (29)	185 (20)	85 (18)	183 (23)	89 (22)
**Use of Bone targeted therapy**, *n° (%)*						
**No**	924 (97)	522 (94)	828 (89)	420 (90)	724 (90)	362 (90)
**Yes**	31 (3)	32 (6)	105 (11)	48 (10)	82 (10)	39 (10)

PLAC, placebo; PSA, Prostate-specific antigen; PSA-DT, PSA doubling time; LN, lymph nodes; PS, performance status; ECOG, Eastern Cooperative Oncology Group; n.s., not specified.

*Follow-up is updated to final analyses of OS (13–15).

### Design and Baseline Characteristics of the Included Studies

Three studies that met the inclusion criteria were included in this analysis (**Table 1**). A total of 4117 high-risk M0 CRPC patients were evaluated: 2694 cases were in the nHT arm (i.e. received the experimental drug plus on-going ADT), and 1423 cases were in the control arm (i.e. received the matched placebo plus on-going ADT). The enrollment of patients was performed between 2013 and 2018. Study design and inclusion criteria were similar among the studies. All the studies were international, randomized, double-blind, placebo-controlled, phase III trials. ADT was continued throughout the trial in all the studies. Based on updated data from the most recent publications on final analyses, patients were followed for a median of 29 to 52 months. As experimental drug, in 1 study was administered Apalutamide 240 mg once daily (806 cases) (11), in 1 Darolutamide 600 mg twice daily (955 cases) (12), and in 1 Enzalutamide 160 mg once daily (933 cases) (10). Patients baseline characteristics were similar among the studies, though with few subtle differences (**Table 1**). Patients in the experimental arm of SPARTAN trial showed a slightly lower median total PSA, compared with PROSPER and ARAMIS trials; patients in the experimental arm of PROSPER trial had a lower median PSA-DT, as well as a higher proportion of patients with PSA-DT ≤ 6 months and a higher percentage of patients with a better PS (ECOG= 0), when compared with PROSPER and ARAMIS trials. Median time from initial diagnosis was shorter in the ARAMIS compared with SPARTAN trial (86.2 versus 95.4 months, respectively); PROSPER trial did not report this data.

### MFS and Time to PSA Progression Analyses

**Figure 1:**
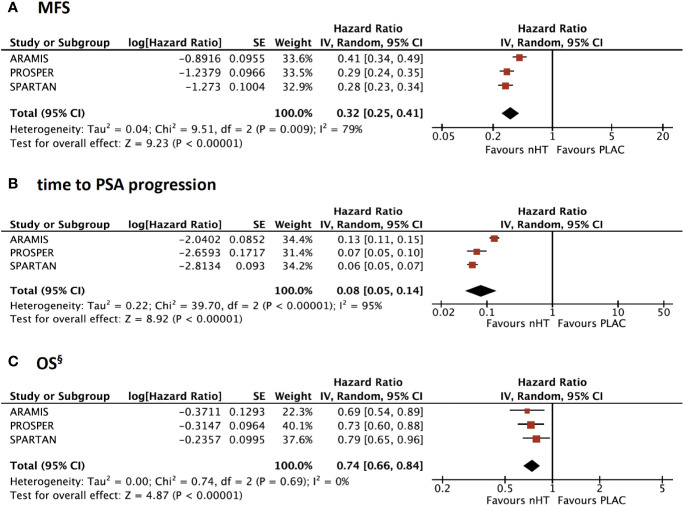
Forest plots reporting pooled survival outcomes from the 3 included studies. **(A)** Metastasis-free survival (MFS); **(A)** time to Prostate-specific antigen (PSA) progression; **(C)** overall survival (OS). ^§^ OS data are updated to final analyses with mature OS data. [CI, confidence interval; nHT, novel hormonal therapy; PLAC, placebo].

MFS was the primary endpoint in all included trials. The pooled analysis showed a significantly better MFS with nHT than with placebo (HR= 0.32, 95%CI= 0.25–0.41) (**Figure 1A**). Similarly, time to PSA progression was significantly improved with nHT compared to placebo (HR= 0.08, 95%CI= 0.05–0.14) (**Figure 1B**).

### OS Analysis: Updated and Mature Results From Final Analyses

At the primary analyses, OS data were immature for all the trials since median OS was not reached in either treatment groups. OS data from final analyses of the included trials are summarized in **Table 2**. The median follow-up was 29 to 52 months; ARAMIS trial showed a shorter follow up time (29 months) when compared to PROSPER and SPARTAN trials (48 and 52 months, respectively). Death events occurred less frequently in ARAMIS trial (n= 148, 15%) than PROSPER and SPARTAN trials (n= 288, 31% and n= 274, 34%, respectively). According to the pooled analysis with updated and mature OS data, OS was significantly improved with nHT compared to placebo (HR= 0.74, 95%CI= 0.66–0.84) (**Figure 1C**). Moreover, nHT significantly improved OS over placebo across all pre-specified subgroups (**Figure S3**, **Supplementary Material**). Subgroup analysis revealed a greater OS benefit in patients with PSA-DT >6 months than ≤6 months (HR= 0.69 versus HR= 0.75), ECOG 0 than 1 (HR= 0.70 versus HR= 0.80), N1 disease than N0 (HR= 0.61 versus HR= 0.78), and in those receiving bone-targeted therapy (HR= 0.65 versus HR= 0.74), and a comparable OS by number of prior HT (HR= 0.75 versus HR= 0.76, for HT= 1 and ≥2); yet, differences between pre-specified subgroups were not significant (all p> 0.05) (**Figures S3 A–E**).

**Table 2:** Overall Survival (OS) data from the final analyses of the three included trials.

Variable	ARAMIS (14)		PROSPER (15)		SPARTAN (13)	
	(n= 1509)		(n= 1401)		(n= 1207)	
**Arm**	DAROLUTAMIDE	PLAC	ENZALUTAMIDE	PLAC	APALUTAMIDE	PLAC
**Patients**, *n°*	955	554	933	468	806	401
**Death events**, *n° (%)*	148 (15)	106 (19)	288 (31)	178 (38)	274 (34)	154 (38)
**Median OS**, *months (95%CI)*	n.s.	n.s.	67.0 (64.0-NR)	56.3 (54.4–63.0)	3.9 (61.2-NR)	59.9 (52.8-NR)
**HR for OS** *, (95%CI) p*	0.69 (0.53–0.88) 0.003		0.73 (0.61–0.89) 0.001		0.78 (0.64–0.96) 0.016	
**Median follow-up**, *months*	29.0		48.0		52.0	

PLAC, placebo; OS, overall survival; n.s., not specified; NR, not reached; HR, hazard ratio; CI, confidence interval.

### Stratified AEs Analysis

**Figure 2:**
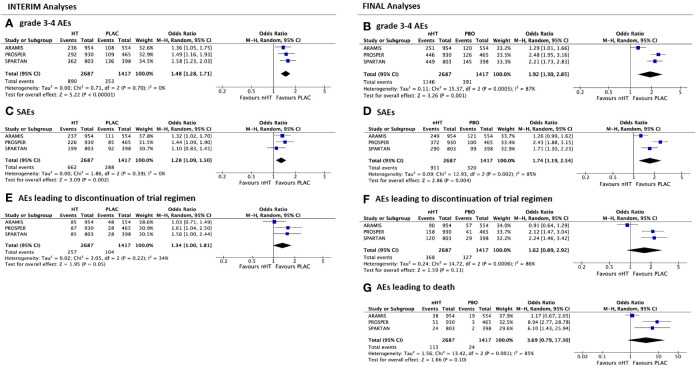
Forest plots reporting pooled safety outcomes from both interim and final analyses of the 3 included studies. Grade 3–4 adverse events (AEs) from interim **(A)** and final analyses **(B)**; serious AEs (SAEs) from interim **(C)** and final analyses **(D)**; AEs leading to discontinuation of trial regimen from interim **(E)** and final analyses **(F)**; AEs leading to death from final analyses **(G)**. [CI, confidence interval; nHT, novel hormonal therapy; PLAC, placebo].

**Figure 3:**
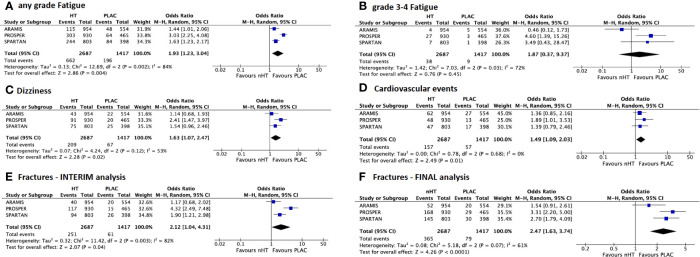
Forest plots reporting pooled safety outcomes from interim analyses of the 3 included studies. **(A)** any grade and **(B)** grade 3–4 fatigue; **(C)** dizziness; **(D)** cardiovascular events; **(E)** fractures from interim analysis; **(F)** fractures from final analysis. [CI, confidence interval; nHT, novel hormonal therapy; PLAC, placebo].

**Figure 2** shows the comparison of AEs reported by both interim (**Figures 2A, C, E**) and final (**Figures 2B, D, F, G**) safety analyses of the included trials. Overall, the nHT arm was significantly associated with higher rates of AEs, when compared with the placebo arm. According to the pooled analysis of data from the safety final analyses of the three trials, the nHT arm was associated with a higher likelihood of experiencing grade 3–4 AEs, SAEs, AEs leading to discontinuation of trial regimen, and AEs leading to death than placebo (OR= 1.92, 95%CI= 1.30–2.85, OR= 1.748, 95%CI= 1.19–2.54, OR= 1.62, 95%CI= 0.89–2.92, and OR= 3.69, 95%CI= 0.79–17.30, respectively) (**Figures 2A–D**). Since published final analyses did not provide sufficient and consistent data to accomplish updated comparisons for all specific types of AEs, rates of specific types of AEs were assessed with data from the safety interim analyses (with the exception of fracture events, which are updated to final analyses). The likelihood of any grade and grade 3–4 fatigue was increased in the nHT arm than in the placebo arm (OR= 1.93, 95%CI= 1.23–3.04, and OR= 1.87, 95%CI= 0.37–9.37, respectively) (**Figures 3A, B**). Similarly, dizziness, cardiovascular events and fractures occurred more often with nHT than with placebo (OR= 1.63, 95%CI= 1.07–2.47, OR= 1.49, 95%CI= 1.09–2.03, and OR= 2.47, 95%CI= 1.63–3.74, respectively) (**Figures 3C–F**).

### Comparison of the Placebo Arms of the Included Studies: Baseline Characteristics, Survival, and Safety Data

Patients baseline characteristics of the placebo arms included in the 3 studies are presented in **Table S2**, **Supplementary Material**. A slightly higher percentage of patients in the PROSPER trial had a shorter PSA-DT of ≤ 6 months (77%), compared to ARAMIS and SPARTAN trial (67% and 71%, respectively), as well as a lower median PSA-DT value at baseline (3.6 versus 4.7 and 4.5, respectively). Regarding lymph nodes status, a higher percentage of N1 patients in the ARAMIS trial was reported, when compared to SPARTAN trial (29% versus 16%); PROSPER trial did not report this data. A worse PS was reported in the placebo arm of ARAMIS trial, with 29% of patients having an ECOG= 1, compared to 18% and 22% for PROSPER and SPARTAN trials, respectively. The use of bone targeted therapy was similar for PROSPER and SPARTAN trials (both 10% of patients), while was slightly lower for ARAMIS (6%).

**Figure 4:**
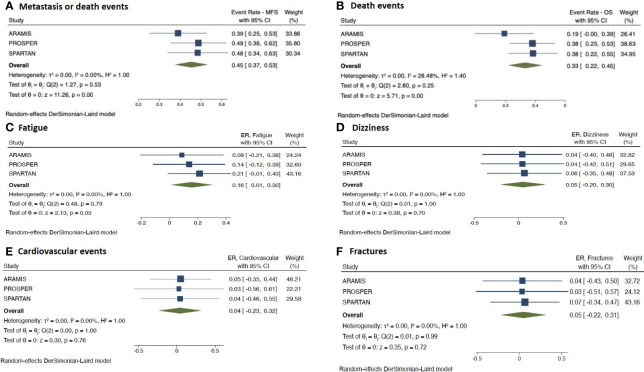
Forest plots reporting pooled survival and safety outcomes from the sole placebo arms of the 3 included studies. **(A)** metastasis or death events; **(B)** death events; **(C)** fatigue; **(D)** dizziness; **(E)** cardiovascular events; **(F)** fractures. [ER, event rate; CI, confidence interval; nHT, novel hormonal therapy; PLAC, placebo].

With regards to survival outcomes, ERs comparing patients in the sole placebo arms showed similar results for metastasis or death events for PROSPER and SPARTAN trials (ER= 0.49, 95%CI= 0.36–0.62 and ER= 0.48, 95%CI= 0.34–0.63, respectively), while ARAMIS showed a lower rate (ER= 0.39, 95%CI= 0.25–0.53) (**Figure 4A**). Similarly, ERs for death events were comparable between PROSPER and SPARTAN trials (ER= 0.38, 95%CI= 0.23–0.53 and ER= 0.38, 95%CI= 0.22–0.55, respectively), whereas ARAMIS showed a lower rate (ER= 0.19, 95%CI= 0.00–0.39) (**Figure 4B**).

With respect to safety outcomes, there was a significant difference in ERs for fatigue reported by the trials (p=0.03) (**Figure 4C**). Fatigue was less common in the ARAMIS arm, showing the lowest ER of 0.09 (95%CI –0.21 to 0.38), when compared with the arms from PROSPER and SPARTAN trials (ER=0.14, 95%CI −0.12 to 0.39 and ER=0.21; 95% CI, −0.01 to 0.43, respectively). On the contrary, placebo arms did not significantly differ in ERs for other analyzed AEs (i.e. dizziness, cardiovascular events and fractures) (all p> 0.05) (**Figures 4D–F**).

### Second-Line Therapies Analysis

**Table 3** summarizes second-line therapies data updated to final analyses reported by the included trials. A higher percentage of patients in the SPARTAN trial received a second line therapy (48%), when compared to ARAMIS and PROSPER trials (15 and 33%, respectively). Chemotherapy with Docetaxel was the most common treatment used in both ARAMIS and PROSPER trials (58 and 60% of patients, respectively), whereas nHT (with either Abiraterone acetate or Enzalutamide) was the most frequent in SPARTAN trial (88% of patients); of note, only 9% of patients in the SPARTAN trial received Docetaxel as second-line treatment. SPARTAN was the sole trial reporting data on second progression-free survival (PFS) (defined as the time from randomization to investigator-assessed disease progression during the first subsequent treatment for mCRPC or death from any cause). At the final analysis, Apalutamide significantly improved second PFS over placebo (HR=0.55, 95%CI −0.46 to −0.66), with an extension of median second PFS of 14.4 months (55.6 months with Apalutamide versus 41.2 months with placebo). Data regarding second PFS were not evaluated in ARAMIS and PROSPER trials (**Table 3**).

**Table 3:** Second line therapies data from the final analyses of the three included trials.

Variable	ARAMIS		PROSPER		SPARTAN	
	(DAROLUTAMIDE, n= 955)		(ENZALUTAMIDE, n= 933)		(APALUTAMIDE, n= 806)	
	INTERIM analysis (12)	FINAL analysis (14)	INTERIM analysis (10)	FINAL analysis (15)	INTERIM analysis (11)	FINAL analysis (13)
**Patients receiving subsequent therapies**, *n° (%)*	100 (11)	141 (15)	138 (15)	310 (33)	165 (21)	386 (48)
**Type of subsequent therapies**, *n° (%)*						
**- DOCETAXEL**	- 49 (49)	- 82 (58)	- 37 (27)	- 185 (60) ^	- 15 (9)	- 33 (9)
**- HT^§^**	- 31 (31)	- 57 (41)	- 52 (38)	- 196 (63)	- 145 (88)	- 314 (81)
**- other^$^**	- 13 (13)	- 2 (1)	- 49 (35)	- 74 (14)	- 5 (3)	- 39 (10)
	*FINAL analysis* (14)		*FINAL analysis* (15)		*FINAL analysis* (13)	
Second progression events^*^, *n° (%)*	not evaluated		not evaluated		319 (40)	
**Median second PFS**, *months*	not evaluated		not evaluated		55.6	
**HR for second PFS** *(95%CI)*	not evaluated		not evaluated		0.55 (0.46–0.66)	
**Median follow-up**, *months*	29.0		48.0		52.0	

HT, hormonal therapy; n.s., not specified; PFS, progression-free survival; HR, hazard ratio; CI, confidence interval.

^§^ includes: ENZALUTAMIDE or ABIRATERONE ACETATE plus PREDNISONE; ^$^ includes other therapies such as CABAZITAXEL, BICALUTAMIDE. ^*^ defined as progression on or after the first subsequent therapy or death.

^^^% are based on the number of patients who received at least one antineoplastic agent after discontinuation of the trial regimen.

## Discussion

The treatment scenario of high-risk M0 CRPC cases has recently and deeply changed, with the shift from the sole on-going ADT to the addition of nHT to on-going ADT. Approval was based on data from the three RCTs: ARAMIS, PROSPER, and SPARTAN, in which Darolutamide, Enzalutamide, and Apalutamide improved MFS over placebo. The pooled benefit in MFS of nHT over placebo was seen in the overall population and analyzed in subgroups analyses; MFS was improved with a greater extent in men with ECOG 0 versus 1, yet no differences were found according to PSA-DT and the use of bone-targeted therapy (18–20).

At this primary analysis, although OS data consistently favored nHT over placebo in all the mentioned trials, the results with respect to OS did not meet the criteria for significance. Recent meta-analyses showed that nHT prolonged OS in a statistically significant manner, yet at that time median OS -still not reached in all experimental arm- and the short follow-up precluded from definitive conclusions (18–20). Results from the prespecified OS final analyses of the 3 trials were presented at the 2020 ASCO Annual Meeting, and then recently published (13–15). To the best of our knowledge, the present work is the first literature-based meta-analysis evaluating results of the final analyses with respect to OS of the three included RCTs.

After a median follow-up of 29 to 52 months, our updated pooled results demonstrated a reduction in death in 26% of patients (HR= 0.74, 95%CI = 0.66–0.84). The pooled analysis revealed the absence of heterogeneity among the studies. However, ARAMIS trial reported the lowest rate of death events in the nHT arm (15% versus 31% and 34% for PROSPER and SPARTAN, respectively), yet it had the shortest follow-up time (29 months versus 48 and 52 months for PROSPER and SPARTAN, respectively). The analysis of the variance distribution of survival data in the sole placebo arms of the trials showed a similar trend. Indeed, ERs for death events were comparable between PROSPER and SPARTAN trials (ER= 0.38 for both) and higher than that ARAMIS showed a lower rate (ER= 0.19). Analysis according to pre-specified subgroups did not show differences by PSA-DT, ECOG score, use of bone-target therapy, LN status and number of prior HT. Indeed, although results revealed a greater OS benefit in groups of patients (i.e. PSA-DT >6 months, ECOG= 0, N1 disease and the use of concomitant bone-targeted therapy), differences were not statistically significant. Therefore, further research is warranted to better define subgroups of patients who will benefit most from nHT.

In view of the long-term treatment with these agents in asymptomatic patients, safety profile covers a pivotal role in the treatment decision-making with these novel compounds.

Overall, patients receiving nHT were more likely to experience AEs, when compared with those receiving the sole on-going ADT. As expected, the long-term analysis (median follow-up of 29 to 52 months) showed a worse safety profile with nHT than the interim analysis (median follow-up of 15 to 20 months). The pooled OR for grade 3–4 AEs increased from 1.48 on interim analysis to 1.92 on final analysis. Of note, ORs in ARAMIS trial remained stable during this time frame, while increased in PROSPER and SPARTAN trials (from 1.49 to 2.48, and from 1.58 to 2.21, respectively). Similarly, the pooled OR for SAEs increased from 1.28 on interim analysis to 1.74 on final analysis; ORs in ARAMIS trial were stable, whereas increased in PROSPER and SPARTAN trials (from 1.44 to 2.43, and from 1.10 to 1.71, respectively). PROSPER trial showed the highest OR increase and value for both grade 3–4 AEs and SAEs, suggesting a higher risk of toxicity at long-term analysis; of note, among patients receiving nHT, PROSPER trial had a higher percentage of patients with a better PS (ECOG= 0) at baseline, when compared with PROSPER and ARAMIS trials. Despite the worse PS showed at baseline in patients receiving Darolutamide compared to other nHT, ARAMIS trial was associated with a more favorable long-term safety profile. Although it reported the shortest follow-up time (29 months), the safety profile appeared to be stable over the time (interim versus final analyses).

With regards to specific types of AEs, rates differed among the evaluated drugs. OR for any grade fatigue on pooled analysis was 1.93; patients in the PROSPER trial experienced more events than those in the ARAMIS and SPARTAN trials (OR 3.03 versus 1.44 and 1.63, respectively). Similarly, patients in the PROSPER trial were more likely to experience dizziness and cardiovascular events (OR 2.41 and 1.89, respectively) than ARAMIS and SPARTAN trials. Concerning fractures, at the interim analyses PROSPER trial showed a somewhat surprisingly higher risk of events (OR= 4.32), than ARAMIS and SPARTAN trials (OR= 1.17 and 1.90, respectively). At final safety analyses, although Enzalutamide was associated with the highest risk among nHT, the risk decreased (OR= 3.31), nuancing the difference among the trials (OR= 1.54 with Darolutamide and OR= 2.70 with Apalutamide).

The difference in safety profiles showed by these trials could be explained by the different structures and mechanisms of action of these novel agents. While Apalutamide and Enzalutamide are androgen receptor inhibitors, Darolutamide is an androgen receptor antagonist. Due to a distinct structure, the latter assures a low penetration of the blood-brain barrier as well as e low binding affinity for κ-aminobutyric acid type A receptors (21, 22). Indeed, data from the final analysis of the ARAMIS trial confirmed the low potential for central nervous system (CNS)-related effects expected with Darolutamide (14). This aspect might be especially important in frail patients, for whom possible CNS-related AEs should be taken into account for assessing the risk-benefit balance of treatment utilization. Although patients in the PROSPER and SPARTAN trials reported higher incidences of CNS-related AEs than those in the ARAMIS, heterogeneous duration of treatment and follow-up could have directly affected these incidences. Moreover, it should be underlined that grade 3–4 CNS-related AEs rates occurred in <1% of patients in all trials. To explore other possible explanation for this difference in toxicity, we evaluated the sole placebo arms of the trials in terms of variance distribution of the AEs rates. Placebo arms significantly differed only in ERs for fatigue, yet not for other analyzed AEs.

Given the use of these agents in asymptomatic patients, health-related quality of life (HRQoL) is a main performance measure - in addition to survival data - that should be taken into account when deciding among treatments. Indeed, HRQoL covers a main role, providing insights into the impact of treatments on patients’ daily life, in terms of both physical and psychological wellbeing (23). Data from the SPARTAN trial demonstrated that HRQoL was not impaired with Apalutamide treatment, and that HRQoL deterioration was more apparent in the placebo group (24). In the PROSPER trial, Enzalutamide showed to increase the time to deterioration in HRQoL, when compared with placebo (25). According to a recent anchored matching-adjusted indirect comparison (MAIC) study, the probability of a better HRQoL with Apalutamide versus Enzalutamide was 73.1% (26). Data from the primary analysis of ARAMIS trial revealed similar QoL scores between Darolutamide and placebo groups, with scores consistently favoring Darolutamide - yet the clinically meaningful thresholds were not reached (12). Since HRQoL is of pivotal importance for patients’ care, better exploring this aspect still represent an area of unmet medical need to guide more informed treatment decisions.

About one-third of patients with M0 CRPC will develop visible bone metastases within 2 years. Currently, there are multiple available therapies for men with mCRPC (i.e. Docetaxel, Abiraterone/Prednisolone, Enzalutamide, Cabazitaxel and Radium–223), and despite the importance of sequencing systemic therapy in mCRPC has already been acknowledged, the optimal strategy of sequencing remains a major challenge. Indeed, selection of treatment for mCRPC is multifactorial and, among other factors, type of previous treatment (e.g. known cross resistance between androgen receptor targeted agents), quality of response and pace of progression on previous treatment have a main role (6, 27). Therefore - and especially after systemic therapies have been moved earlier in the treatment scenario of PC - providing data on response and progression on second-line therapies would be of particular clinical value for accurately managing PC patients over the time.

Unfortunately, only SPARTAN trial provided survival data on second-line therapies (i.e. second PFS), and currently no other data are available to help set the proper sequencing of therapeutic agents, suggesting further research in this field is required. However, results from SPARTAN trial were promising, showing that second progression or death events occurred in 15% of patients receiving Apalutamide, and that this drug extended median second PFS by 14.4 months versus placebo; the HR for second PFS with Apalutamide was reduced by 45% versus placebo (**Table 3**) (13). Owing to the lack of data from other trials, we are not able to make a comparison among different nHT.

In conclusion, to date, main limitations that may affect an optimal treatment decision-making with these novel compounds – and that should represent a field for further research, could be summarized as follow: (I) the lack of comparable HRQoL data; (II) heterogeneous follow-up period; (III) scarce survival data on second-line therapies.

Moreover, it is important to underline that in all the available trials the M0 status was assessed by conventional scans (i.e. computed tomography (CT) and bone scans). According to recent publications, it is reasonable to speculate that with more sensitive imaging modalities (e.g. PSMA PET/CT or whole-body magnetic resonance imaging (MRI)) more patients are expected to be diagnosed with early mCRPC (28), suggesting this setting is expected to evolve in the near future.

## Conclusion

According to the available evidence, nHT have shown to improve MFS as well as - according to final analyses - OS over placebo in the setting of high-risk M0 CRPC. Owing to the importance of sequencing systemic therapy in CRPC, the lack of survival data regarding second-line therapies remains a major issue. The long-term analysis showed a worse safety profile with nHT than the interim analysis, whit distinct profiles among different nHT. Moreover, phase IV trials evaluating nHT in a real-world setting would be of particular clinical value to help guide proper treatment choices in these patients. Lastly, whether the use of novel imaging modalities will change treatment decision in this setting represents an open question for the near future.

## Data Availability Statement

The original contributions presented in the study are included in the article/**Supplementary Material**. Further inquiries can be directed to the corresponding author.

## Author Contributions

Conceptualization: MM and AS. Methodology: VF and GMB, Software: UGF, Formal analysis: MM and FDG. Investigation: VF and GDV. Resources: MF and AP. Data curation: SS. Writing—original draft preparation: MM. Writing—review and editing: AS and EDB. Supervision: AS. All authors contributed to the article and approved the submitted version.

## Conflict of Interest

The authors declare that the research was conducted in the absence of any commercial or financial relationships that could be construed as a potential conflict of interest.
