# Supplementary material for: A Systematic Review and Meta-Analysis of Randomized Controlled Trials With Novel Hormonal Therapies for Non-Metastatic Castration-Resistant Prostate Cancer: An Update From Mature Overall Survival Data
Source: Front Oncol. 2021 Jun 8;11:700258. doi: 10.3389/fonc.2021.700258 (PMC8217817; doi:10.3389/fonc.2021.700258)

Supplementary Material

**Supplementary Table S1.** Inclusion criteria following the Population, Intervention, Comparison, Outcomes and Study design (PICOS) method.

| Population | men diagnosed with high-risk (i.e. PSA-DT ≤ 10 months) M0 CRPC |
| --- | --- |
| Intervention | nHTs (Apalutamide, Enzalutamide, or Darolutamide) *plus* on-going ADT |
| Comparisons | placebo arm (i.e. received the sole ADT) |
| Outcomes | - MFS - OS - time to PSA progression - second-line therapies data - AEs (overall AEs and grade 3-4 AEs) including all AEs, serious AEs, AEs leading to discontinuation of trial regimen, AEs leading to death, fatigue, dizziness, cardiovascular events, and fractures - impact of variables on updated OS data: - PSA-DT (≤ *versus* >6 months) - PS (ECOG score 0 *versus* 1) - use of bone-targeted therapy (yes *versus* no) - LN status (N0 *versus* N1) - prior HT (1 *versus* ≥2) |
| Study design | randomized controlled trials |

M0 CRPC= non-metastatic castration-resistant prostate cancer; ADT= androgen deprivation therapy; nHTs= novel hormonal therapies; MFS= metastasis-free survival; OS= overall survival; PSA= prostate-specific antigen; AEs= adverse events; PSA-DT= PSA doubling time; ECOG= Eastern Cooperative Oncology Group; PS= performance status; LN= lymph lodes.

**Supplementary Table S2. Patients baseline characteristics of the sole placebo arms of the 3 included studies** [number of cases (%), and median (range)].

| *Variable* | **ARAMIS** [12] | **PROSPER** [10] | **SPARTAN** [11] |
| --- | --- | --- | --- |
| **Patients**, *n°* | 554 | 468 | 401 |
| **Age** (years)*, median (range)* | 74 (50-92) | 74 (53-92) | 74 (52-97) |
| **Follow-up *** (months)*, median* | 29.0 | 48.0 | 52.0 |
| **Time from initial diagnosis** (months)*, median* | 84.2 | n.s. | 94.2 |
| **total PSA level** (ng/mL)*, median (range)* | 9.7 (1.5-885.2) | 10.2 (0.2-467.5) | 8.0 |
| **Testosterone level** (nmol/L)*, median (range)* | 0.6 (0.2-7.3) | n.s. | 0.8 (0.3-2.8) |
| **PSA-DT***, n° (%)*  ≤ 6 months  > 6 months  *median* (ng/mL) | 371 (67)  183 (33)  4.7 | 361 (77)  107 (23)  3.6 | 284 (71)  117 (29)  4.5 |
| **LN status**, *n° (%)*  **N0**  **N1** | 396 (71)  158 (29) | n.s. | 336 (84)  65 (16) |
| **ECOG PS score**, *n° (%)*  **0**  **1** | 391 (71)  163 (29) | 382 (82)  85 (18) | 311 (78)  89 (22) |
| **Use of Bone targeted therapy**, *n° (%)*  **No**  **Yes** | 522 (94)  32 (6) | 420 (90)  48 (10) | 362 (90)  39 (10) |

PSA= Prostate-specific antigen; PSA-DT= PSA doubling time; LN= lymph nodes; PS= performance status; ECOG= Eastern Cooperative Oncology Group: n.s.= not specified.

* Follow-up is updated to final analyses of OS [13-15]

**Supplementary Figure S1**. PRISMA flow diagram.


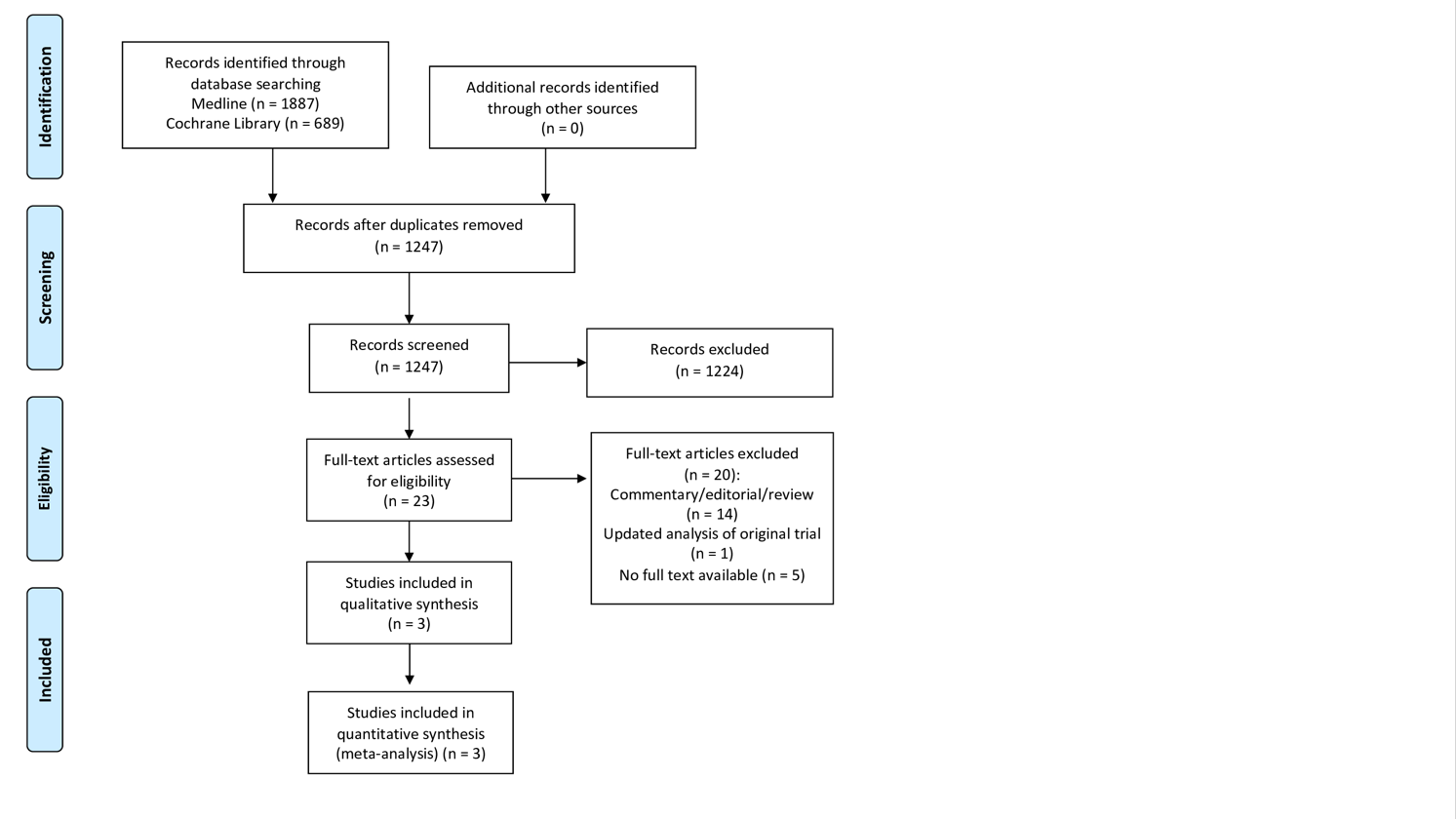


**Supplementary Figure S2**. Risk of bias (RoB) for all included studies was evaluated using the Review Manager tool for the assessment of the methodological quality of trials.


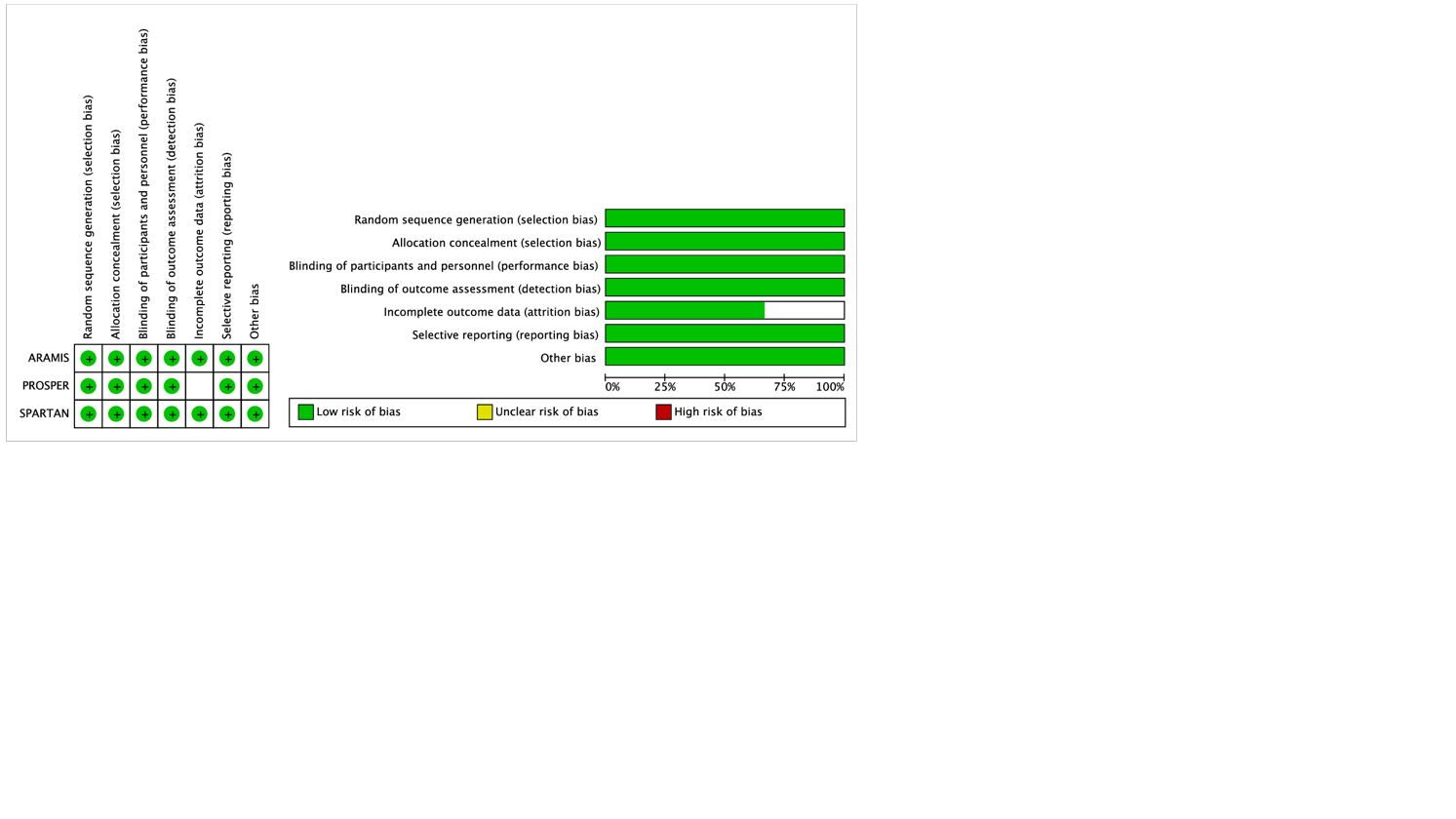


**Supplementary Figure S3**. Forest plots reporting pooled overall survival (OS) outcomes by patient subgroups: **(A)** PSA-DT, <= 6 versus > 6 months; **(B)** ECOG, 0 versus 1; **(C)** use of bone targeted therapy, no versus yes; **(D)** LN status, N0 versus N1; **(E)** number of prior HT, 1 versus >=2.

[PSA-DT= Prostate-specific antigen doubling time; ECOG= Eastern Cooperative Oncology Group; LN= lymph nodes; HT= hormonal therapy; CI= confidence interval; nHT= novel HT; PLAC= placebo]


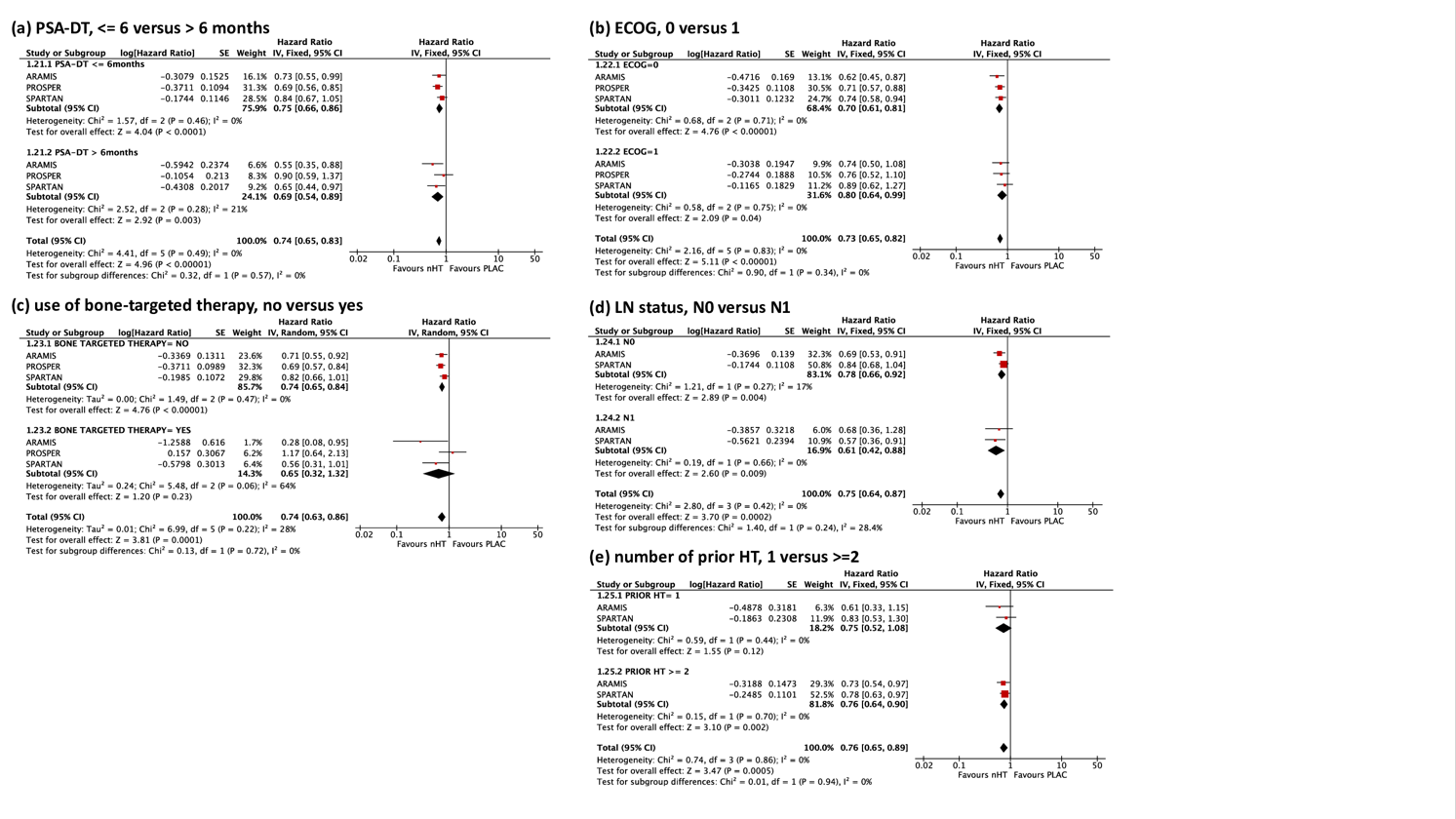

Supplement: Supplementary file 1 [file DataSheet_1.docx]
